# TPV: A New Insight on the Rubber Morphology and Mechanic/Elastic Properties

**DOI:** 10.3390/polym12102315

**Published:** 2020-10-10

**Authors:** Cindy Le Hel, Véronique Bounor-Legaré, Mathilde Catherin, Antoine Lucas, Anthony Thèvenon, Philippe Cassagnau

**Affiliations:** 1 Univ-Lyon, Université Claude Bernard Lyon 1, Ingénierie des Matériaux Polymères, CNRS, UMR 5223, 15 Bd Latarjet, 69622 Villeurbanne CEDEX, France; cindy.le-hel@univ-lyon1.fr (C.L.H.); veronique.bounor-legare@univ-lyon1.fr (V.B.-L.); mathilde.catherin@univ-lyon1.fr (M.C.); 2 Hutchinson, Centre de Recherche, Rue Gustave Nourry-B.P. 31, 45120-Chalette-sur-Loing, France; antoine-p.lucas@hutchinson.com (A.L.); anthony.thevenon@hutchinson.com (A.T.)

**Keywords:** thermoplastic vulcanizates, morphology, compression set

## Abstract

The objective of this work is to study the influence of the ratio between the elastomer (EPDM) phase and the thermoplastic phase (PP) in thermoplastic vulcanizates (TPVs) as well as the associated morphology of the compression set of the material. First, from a study of the literature, it is concluded that the rubber phase must be dispersed with a large distribution of the domain size in the thermoplastic phase in order to achieve a high concentration, i.e., a maximal packing fraction close to ~0.80. From this discussion, it is inferred that a certain degree of progress in the crosslinking reaction must be reached when the thermoplastic phase is melted during mixing in order to achieve dispersion of the elastomeric phase in the thermoplastic matrix under maximum stress. In terms of elasticity recovery which is measured from the compression set experiment, it is observed that the crosslinking agent nature (DCP or phenolic resin) has no influence in the case of a TPV compared with a pure crosslinked EPDM system. Then, the TPV morphology and the rubber phase concentration are the first order parameters in the compression set of TPVs. Finally, the addition of carbon black fillers leads to an improvement of the mechanical properties at break for the low PP concentration (20%). However, the localization of carbon black depends on the crosslinking chemistry nature. With radical chemistry by organic peroxide decomposition, carbon black is located at the interface of EPDM and PP acting as a compatibilizer.

## 1. Introduction

Thermoplastics vulcanizates (TPVs) are a family of polymer blends with a range of original and varied physical, mechanical, and processing properties. A TPV formulation generally consists of an elastomer phase, a thermoplastic phase, a crosslinking agent, inorganic fillers, plasticizers, stabilizers, and compatibilizing agents. The most developed TPVs to date are those based on polypropylene (PP) and a copolymer of ethylene-propylene-diene monomer (EPDM), but there are some examples in which the elastomer phase consists of another material (poly(butadiene-acrylonitrile), polyisoprene, brominated isoprene, silicone, etc.) [1].

The unique and specific character of TPVs is that the elastomer phase is crosslinked under flow during the mixing process. This leads to significant morphological changes, in particular phase inversion, due to the rapid and significant change in the viscoelasticity of the elastomer, from viscoelastic liquid to viscoelastic solid. The formation of an elastic rubber phase dispersed in the thermoplastic matrix allows the material to present its original properties. Finally, the mechanical properties of a TPV are close to those of a crosslinked rubber, while maintaining the processing characteristics of thermoplastics.

The properties of TPVs depend on various factors such as the formulation (polymers, fillers, plasticizers, crosslinking agent), the morphologies (dispersed and/or continuous phases), and the processing methods (batch and/or continuous processes). The final morphology of these reactive blends depends on the concentration and nature of the main constituents, the interface between the polymers, the crystallinity of the thermoplastic phase, the kinetics of crosslinking reaction, the presence of a compatibilizing agent, and so on. In order to control the morphology development throughout the elaboration process, several aspects must be considered: (i) the crosslinking chemistry of the elastomer phase and its consequences on rheology variation, (ii) the compatibility between phases taking into account the aspects of interfacial tension, (iii) the dispersion of low viscosity compounds (crosslinking agents, plasticizers) and the dispersion/localization of fillers in both phases, and (iv) the manufacturing process (batch mixing and/or continuous extrusion) with the associated viscous dissipation phenomena (self-heating).

Consequently, the morphology development in the TPV has attracted a lot of attention in the last decades and the mechanisms of formation of these rubber domains have been extensively studied from experimental [1,2] and theoretical [3] points of view. It is not possible to report and to exhaustively discuss all the papers published in this domain; however, some reviews are of interest [3,4]. The TPVs were developed to replace conventional elastomers, which once crosslinked can no longer be processed, consequently limiting their fields of applications and their recyclability. As pointed out before, TPVs combine both the processing properties of thermoplastics and the elasticity of crosslinked rubbers. In terms of application, the required main property is the elastic recovery, more commonly called compression set (Cs%). The compression set characterizes the ability of a material to recover its initial shape after being submitted to a constant strain. In the standard tests practiced in the industry, a strain of 25% is applied for several hours at a temperature higher than room temperature, in the ASTMD 395 standards (ISO 815-1) the material is compressed during 24 h at a temperature of 100 °C.

In terms of real industrial applications, the objective is to reach the lowest compression set value. Although we cannot obtain the compression set of the pure elastomeric phase due to the plastic deformation of the thermoplastic phase, the tendency is to reach compression set lower than 30%. It is clear that this property is governed by the formulation and the morphology developed during the TPV processing. It is generally believed that a fine dispersed rubber phase is the key to obtain good mechanical properties and high elasticity of TPV products [5]. However, the concentration in the elastomer phase must be as high as possible while keeping a continuous thermoplastic phase for the processing ability.

The question that must be asked then is: what is the highest concentration of the elastomer phase which can be dispersed in a thermoplastic matrix? It is actually common to observe in various publications and industrial TPVs that the elastomer phase concentration is higher than 60% and up to 80% [6,7]. The objective of this paper is to study the evolution of the morphology at this high level of crosslinked elastomer phase and, first, to explain how such a level of concentration can be reached. Next, we study the influence of the concentration of the elastomer phase on the recovery elasticity of these TPVs as well as the influence of the reinforcing fillers such as carbon black. 

### 1.1. TPV: A Highly Filled Thermoplastic Polymer

**Figure 1:**
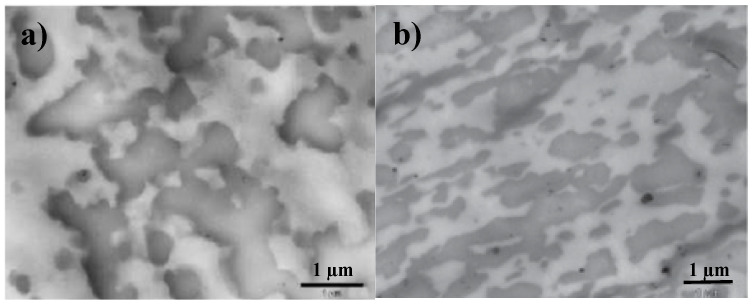
Examples of thermoplastic vulcanizate (TPV) morphology (crosslinked by Resol) from Transmission Electronic Microscopy (TEM) (**a**) PP/EPDM: (30/70) [2] (**b**) PP/EPDM (50/50) [8] with permission from Elsevier.

In molten state, a TPV can be considered as a fluid with a very high solid phase content. Indeed, it can be assumed that once the TPV has been developed, the crosslinked elastomer domains behave as a weakly deformable phase in a shear thinning fluid. In fact, this rubber phase has a shear modulus greater than ~2.10^5^ Pa and behaves as a viscoelastic solid in a suspending viscoelastic liquid. Theoretically, for suspensions, the maximal packing fraction Φ_m_ of randomly dispersed spherical particles is equal to 0.64. That is to say, if the elastomer domains were perfectly spherical, regardless of their size, a TPV could not contain more than 64 % vol of elastomer phase. However, as illustrated in Figure 1, we can observe that this morphology is not spherical and that it has a very poorly defined geometrical shape which looks more like an asteroid (see Figure 1) than a planet. This aspect ratio, difficult to quantify precisely from electronic microscopy analysis, is favorable to increase the maximal packing fraction.

Actually, as it is well known in a suspension of spherical particles, the main strategy for increasing Φ_m_ is to control the particle size distribution. The shape of the particles as well as their aspect is of importance [9], but this influence is strongly dependent on the particles’ structures. This aspect has been well-studied in the case of suspensions, see, for example, some articles on the rheology of concentrated suspensions of arbitrarily shaped factors [10]. 

To increase Φ_m_ for spherical particles, the solution adopted is a bimodal particles population and several models have been developed in this field [11,12]. Bimodal suspensions are characterized in terms of diameter or size ratio λ defined as the ratio between the largest particle diameter (D_L_) to the finest particle diameter (*D*_f_), λ = *D*_L_/*D*_f_ as well as by the volume ratio between each population ξ= Φ_L_/Φ_f_. Generally, it is preconized λ ≈ 8 and ξ = 0.9.

**Figure 2:**
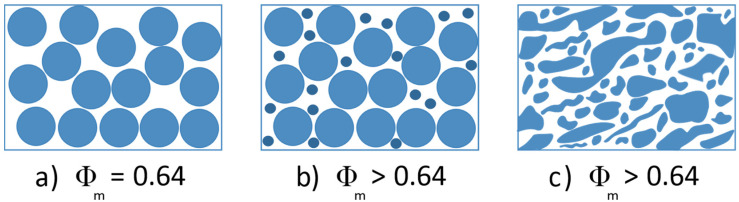
Scheme of different morphologies with the corresponding maximal packing fraction Φ_m_ that can be expected. (**a**) monodispersed spherical system, (**b**) bimodal spherical system, (**c**) broad distribution of ellipsoid shape system.

A bimodal population can be only obtained in the case of theoretical model systems. However, a broad distribution of particle size combined with a more or less ellipsoid shape is obviously favorable to increase Φ_m_. Indeed, on the TPV morphology depicted in Figure 1, a broad distribution of domains size from sub-micron scale to the micro-scale (10 μm) can be observed. Indeed, this specific morphology allows the TPV to reach a concentration in the elastomer phase close to 80% whereas the concentration Φ_m_ = 0.64 cannot be exceeded for spherical particles (Figure 2). 

Finally, do these morphologies information then give us the conditions to elaborate a TPV with the largest possible Φ_m_ i.e., with the smallest thermoplastic phase concentration?

### 1.2. Morphology Development

First, it must be pointed out that most of the time, TPV formulations contain a large amount of oil which can exceed 50 wt %. However, regarding TPVs based on EPDM/PP, this oil is homogeneously distributed between the thermoplastic phase and the EPDM phase. Our latest studies have shown that during the crystallization of PP a part of this oil is expelled and migrates to the elastomer phase which is richer in oil than the thermoplastic phase. However, the global ratio between the elastomer phase (EPDM/oil) and the thermoplastic phase (PP/oil) remains more or less the same. This result has also been demonstrated by the work of Caihong et al. [13]. Thereafter, we will only discuss the solid-elastic elastomer phase and the liquid thermoplastic phase.

Many studies on the development of TPV morphologies have focused on the influence, on the one hand, of the crosslinking reaction on the dispersion of the elastomer phase whose viscoelastic properties change considerably with crosslinking, and, on the other hand, the influence of the shear rate of the mixture on these dispersion mechanisms as well. At the early stage of the processing mixing, there is a blend of two viscoelastic fluids. As the PP is a minor phase with respect to the concentration (<30 wt %), the expected morphology is the dispersion of PP droplets in the EPDM which is governed by the capillary number and Rayleigh instabilities [2]. At the end of the mixing/reaction process, a totally different morphology is observed due to the phase inversion phenomenon induced by the crosslinking reaction. The balance between the crosslinking reaction and mixing intensity is the dominant parameter for the morphology development. If the reaction is faster than the mixing time, the whole material turns into powder due to the macroscopic fragmentation of the EPDM phase. If the reaction is much slower, then the mixing is a priori favorable to control this phase inversion and it is expected to be the right morphology (which does not necessarily mean that the right properties have been achieved). 

However, it is difficult to dissociate the mixing from the chemical reaction due to the fact that in these high viscosity media, the viscous dissipation phenomena are extremely high. Thus, the temperature and therefore the chemical reaction is difficult to control, especially if these reactions are of the radical type and are characterized by high activation energies. In order to dissociate these combining effects, Martin et al. [14] studied the dispersion of pre-crosslinked EPDM samples at different extents of crosslinking in the PP phase. They showed that for an EPDM insoluble fraction lower than 0.70, the PP/EPDM blend behaves, from a dynamic point of view, as a usual blend whose morphology is governed by the mechanisms of break up/coalescence. Even if the elasticity ratio changes drastically, this equilibrium remains governed by the capillary number expressed at the applied shear rate. Beyond this level of elasticity, the morphology is established by the fragmentation of the EPDM phase leading to the phase inversion and finally to the TPV morphology. In order to be able to process TPV with a low concentration (<30 wt %) of the thermoplastic phase, this fragmentation of the EPDM must be as fine as possible with a wide size distribution. 

In order to achieve this fragmentation, it is therefore imperative to apply the highest possible stresses to the EPDM phase. It is necessary to use a thermoplastic phase of high viscosity and therefore high molar mass. However, it can be seen from a mixing torque curve in a batch mixer that the highest stress is obtained during the melting phase of the polymer pellets. This phenomenon is rarely explained in the literature, but it can be stated that it is due to the friction of the PP pellets that are melting, as in a sintering process. This thermoplastic phase melting event is therefore the one that generates the highest stress on the EPDM phase. Consequently, and from a practical point of view, the crosslinking reaction must be advanced enough to undergo the fragmentation of the EPDM phase when the PP melts. 

To conclude, how can one achieve a concentration of the elastomer phase close to 80% while reaching a material with good mechanical properties (elongation at break, for example). On one hand, it is essential to have perfect stress continuity at the interface. In the case of PP/EPDM systems, this stress continuity is favorable due to the low interfacial tension, γ_1-2_ ≈ 0.3 mN/m [15], and the fact that the oil plays the role of the interfacial phase between PP and EPDM [6]. The presence of fillers such as carbon black can also play this role of interfacial compatibility as reported for blend systems [16]. In the case of the TPV, the influence of fillers on the morphology and final properties has not been investigated in the literature, to the best of our knowledge. 

## 2. Experimental

### 2.1. Materials

The experiments were carried out with EPDM Vistalon 8600 (ExxonMobil Chemical, Houston, TX, USA), in which the diene nature is ethylidene norbornene (ENB). The following values of average molar masses were measured by Size Exclusion Chromatography with a conventional calibration in trichlorobenzene at 150 °C: ${\bar{M}}_{n}$ = 31,000 g/mol and ${\bar{M}}_{w}$ = 107,000 g/mol. The molar content of each component in the terpolymer was assessed by ^1^H NMR: 71.6 mol % ethylene, 26.4 mol % propylene and 2.0 mol % ENB. Vistalon 8600 has a density of 0.86 g/cm^3^ and a Mooney viscosity ML_(1+8)_ of 81 at 125 °C. Isotactic PP (PPH 3060, Total Petrochemicals, Courbevoie, France) was also used, with a melt flow index MFI = 1.8 g/10 min at 230 °C/2.16 kg. Its average molar masses are ${\bar{M}}_{n}$ = 72,000 g/mol and ${\bar{M}}_{w}$ = 384,000 g/mol. Additional paraffinic oil (Nypar 330, Nynas, Stockholm, Sweden) was incorporated to mimic industrial compositions. The density of this plasticizer is 0.875 g/cm^3^ at 15 °C. Its proportion in the binary EPDM/plasticizer mixture was set to the industrial standard for most samples, i.e., 122 phr (grams per hundred grams of Vistalon 8600). 

EPDM crosslinking reaction was carried out with either an organic peroxide (Dicumyl peroxide (DCP) 99% purity, Sigma Aldrich, Saint-Louis, MO, USA) or with an octylphenol-formaldehyde resin (Nures 2055, Newport Industries, ShanXi, China) called Resol in the following text. In order to modulate the degree of crosslinking, the EPDM samples were prepared with diverse amounts of the crosslinking agent. The carbon black (CB) N550 (Lehvoss, Cherisy, France) has a specific surface area of 40 m^2^/g and a density of 1.7–1.9 g/cm^3^ at 20 °C.

### 2.2. Samples Preparation

The sample preparation method is based on the one described by Ning et al. [17]. The blends of the polymers, processing oil, and crosslinking system (radical initiator or Resol crosslinker) were prepared in an internal batch mixer (Haake Rheomix 600, Thermo Fisher Scientific). The following protocol was adopted: first, the EPDM, the curing system (Resol or DCP), the carbon black filler (if needed), and the plasticizer were introduced (at time *t* = 0) into the chamber at 60 °C and mixed for 5 min at 50 rpm, and then the PP pellets were introduced at *t* = 5 min and mixed for 5 min. The second phase of the process consisted of an increase of the control temperature up to 180 °C, still at 50 rpm. The dynamic vulcanization occurred during this phase. The time for this mixing process depends on the formulation. The mixing is stopped once the temperature and the torque are stabilized. All this mixing process takes around 20–25 min. 

The samples were compression molded into 2 mm-thick sheets for 10 min at 180 °C for samples cured by peroxide and for 20 min at 200 °C for samples cured by Resol. The formulations, i.e., concentration of the PP, EPDM, oil, curing agent, and of all samples prepared for this study are reported in Table 1.

### 2.3. Compression Set

The elastic capacity of a crosslinked TPV samples to recover its initial size after being subjected to constant deformation for a specified time at a given temperature is evaluated by the compression set test. This test is standardized by ASTMD 395 standards (ISO 815-1). 

The cylindrical sample, 13 mm in diameter and l_0_ = 6 mm thick, is 25% compressed (ε_0_ = 0.25) in an oven for 24 h at 100 °C and then removed and left to cool for 30 min. The compression set is then expressed by:

(1) $$\mathrm{Cs}\;\left(\%\right)=\frac{1}{\varepsilon_{0}}\left(1-\frac{l_{1}}{l_{0}}\right).100$$

l_1_ the final thickness of the sample and $\varepsilon_{0}$ the nominal deformation imposed to 0.25. The residual deformation or elastic recovery of the material can then be determined. The following limits of the compression set data under these experimental conditions (temperature and loading time) are then: Cs = 0%, as l_1_ = l_0_, where the sample shows perfect elastic recovery and Cs = 100%, as l_1_ = l_0_ (1 − ε_0,n_), where the sample reflects no elastic recovery.

### 2.4. Electron Microscopy

#### 2.4.1. Scanning Electron Microscopy (SEM)

Each sample was first cryogenically surfaced by ultramicrotomy, then the EPDM phase was selectively extracted by immersion in THF for 4 days. After drying, the samples were covered with a homogeneous 10 nm copper deposit by plasma metallization. The microscopic observation was performed using a QUANTA 250 SEM at a voltage of 10 kV.

#### 2.4.2. Transmission Electron Microscopy (TEM)

The samples were first ultra-microtomed into thin pieces of about 70 nm in thickness with a Leica UC 7 under liquid nitrogen atmosphere at −160 °C. The lamellae were then deposited on copper grids (Mesh 300). The observation was carried out using a Philips CM 120 TEM transmission electron microscope. The EPDM phase appears dark on the images, while the polypropylene is very light in appearance.

#### 2.4.3. Mechanical Properties

Standard tensile tests were conducted on dumbbell-shaped H2 specimens using a tensile test machine (Shimadzu AGS-X, force sensor: SSM-DAK-5000N) at room temperature. The test speed was kept at 500 mm/min according to ISO 37-2017. The tensile strength, the elongation at break, and the Young modulus were calculated from at least five specimens for each sample and the results were averaged.

## 3. Results and Discussion

**Figure 3:**
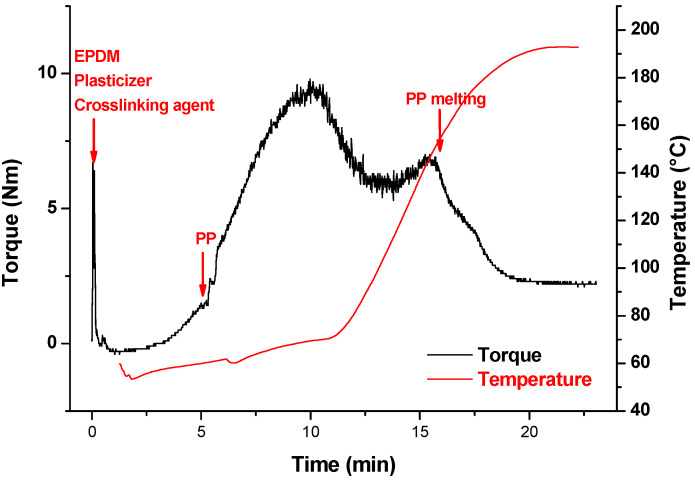
Mixing conditions of TPV in batch mixer. Variation of the torque and temperature versus mixing time. Formulation (phr): EPDM = 100, Oil Plasticizer = 122; PP = 75, DCP = 1. N = 50 rpm.

Figure 3 shows the variation of the torque and temperature versus the mixing time under a rotor speed of 50 rpm. In the first mixing steps, the increase in torque is mainly due to the diffusion and homogeneous mixing of the oil with the EPDM. This increase in torque is also due to the introduction of solid PP pellets which increases the filling rate of the mixer chamber. The blend is finally homogeneous when the torque curve passes through this maximum. Due to the increase of the temperature, the torque decreases as expected after this maximum. However, a second maximum corresponding to the melting temperature of the PP (T ≈ 162 °C) emerges. It is, however, surprising that the melting of the PP leads to an increase in torque as the pellets change from a solid state to a molten liquid state. In fact, this phenomenon is due to interaction/friction (plastic deformation) between the PP pellets and is comparable to a sintering phenomenon. The stress intensity obtained in this way is higher than the stress level in the liquid state. This transition zone during mixing can be then expected to be the most favorable for optimal EPDM phase dispersion during the phase inversion phenomenon. In a quantitative way, the effect of the melt temperature cannot be dissociated from the effect of the shear rate as the temperature of the system results from the viscous dissipation phenomenon. In the case of EPDM crosslinking with a radical initiator, the quantity of free radical generated can be calculated from the thermal kinetic decomposition of DCP according to the following equation:

(2) $$\frac{d\left[\mathrm{DCP}\right]}{\mathrm{dt}}=\;-k_{d}\;\left[\mathrm{DCP}\right]$$

with *k*_d_ the dissociation constant: $k_{d}=A.e^{-\mathrm{Ea}/\mathrm{RT}}$, A the frequency factor: $A=7.47\times 10^{15}{\;s}^{-1}$; and E_a_ the activation energy of the reaction: $E_{a}=153.5\;\mathrm{kJ}/\mathrm{mol}.$

The crosslinking is initiated by the thermal decomposition of the peroxide. Next, the active radicals formed generally react with the elastomer chains by removing hydrogen atoms from the carbon backbone of the polymer, creating highly active radicals on the chain. Finally, crosslinking results by the combination of two macroradicals or by the addition of a macroradical to the unsaturated portion of another major elastomer chain. This produces a carbon–carbon crosslink.

**Figure 4:**
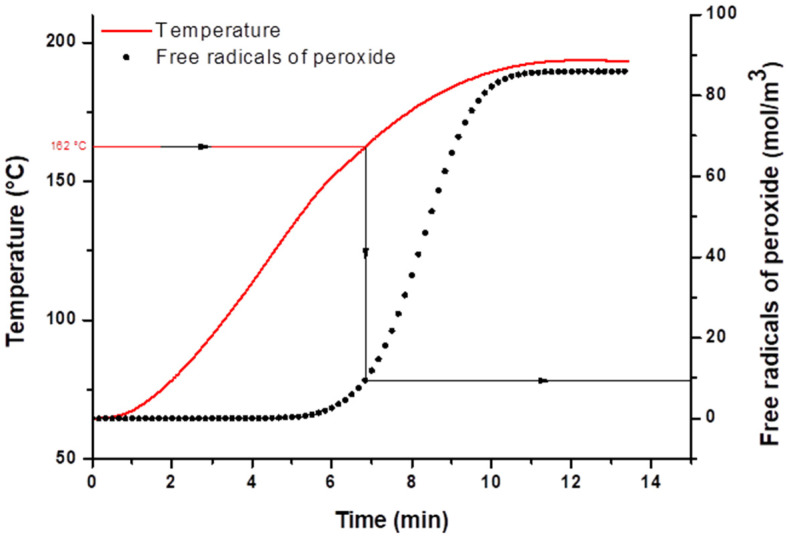
Prediction of free radical concentration generated during mixing in the EPDM phase from thermal dicumyl peroxide (DCP) composition. The temperature curve is from melt temperature recorded during the mixing experiment (Formulation depicted in Figure 3).

As a result of this prediction of free radicals concentration, it can be then observed that the phase inversion, near 160 °C and thus the melting point of the PP, takes place at a concentration of ~10 mol/m^3^ of free radicals created (Figure 4). This result proves that the phenomenon of phase inversion occurs for the early steps of the crosslinking reaction while the total creation of radicals at the end of the mixing process is 83 mol/m^3^. However, in terms of crosslinking density, 10 mol/m^3^ corresponds to a level of crosslinking for an EPDM with a certain level of elasticity which, according to our previous work [18], results in an elastic modulus of 6 × 10^4^ Pa, a swelling rate of 20, and a compression set of pure crosslinked EPDM of 60%. Most importantly, from the point of view of viscoelasticity and deformation of the elastomer phase, this level of crosslinking corresponds to the regime for which the morphology is developed by the erosion and fragmentation of weakly crosslinked elastomers domains as already proven by Martin et al. [2]. The crosslinking kinetics of the type of phenolic resin used in this study is not known in the literature and we are therefore unable to extend this study to TPVs crosslinked with phenolic resin.

**Figure 5:**
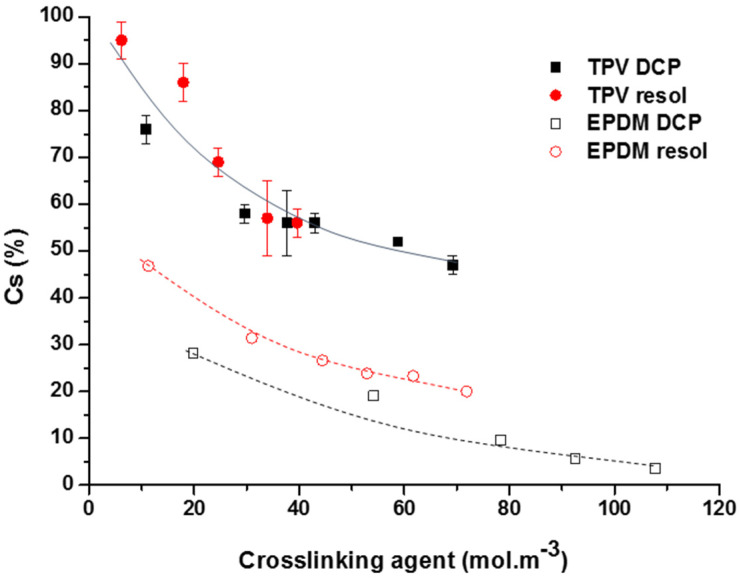
Variation of the compression set versus the molar concentration of the crosslinking agent for TPV with 75 phr of PP. Comparison between the TPV (full symbols) and the neat EPDM systems (open symbols from our previous work [18]).

As we have seen in our previous work [18], the compression set of the elastomeric phase is mainly influenced by the crosslinking chemistry. In fact, better elasticity recovery properties as shown in Figure 5 are obtained with the peroxide radical initiator in regard to the Resol-based crosslinking system because DCP results in a more statistical crosslinking reaction. If we consider that only the elastomeric phase is a major influence on the elasticity recovery of the TPV, we can easily first imagine that the tendency will be similar for a TPV. However, in Figure 5, it can be observed for the TPVs that both crosslinking ways lead to the same trend and that there is not a significant difference between the two in term of compression set. Moreover, it can be clearly observed that the elasticity recovery of TPV is lower (higher Cs) than for neatly crosslinked EPDM. 

For example, we can notice that with around 40 mol·m^−3^ of the crosslinker, in the EPDM matrix, our previous work showed a compression set of ~20% for the system crosslinked by DCP and of ~30% for the system crosslinked by Resol, whereas, in the TPV with this same amount of crosslinking agent, the compression set is around ~60%. It can be then concluded that there are other factors, such as PP concentration and morphology that influence the elasticity recovery of the TPV. 

These observations mean that the addition of the PP has an important influence on the elasticity recovery property of the TPV material. The role of the PP in the TPV is to make it processable like a thermoplastic while maintaining the mechanical properties, for example, elongation at break. Accordingly, the concentration of the PP was decreased from 75 (25 wt %) to 25 phr (10 wt %) in order to study the influence of the thermoplastic phase on the compression set experiments. 

**Figure 6:**
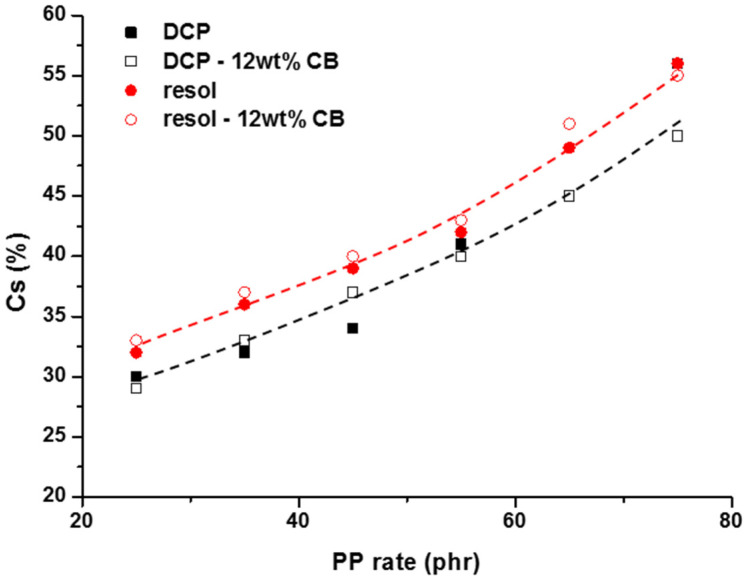
Variation of the compression set (Table 2) versus the PP concentration in the TPV formulation. Amount of chemical reagent in the formulations: 4 phr (DCP) and 7 phr (Resol).

Figure 6 clearly shows that the concentration of PP has a strong influence on the elasticity recovery of TPVs: the less the PP concentration is, the better compression set is. The values of the compression are reported in Table 2. For example, a compression set of 30% is reached for peroxide-based TPV with 25 phr of PP. Overall, from the set of values, it seems that the use of DCP as a radical initiator, compared to the use of the Resol crosslinker, leads to better results in terms of elastic recovery. 

**Figure 7:**
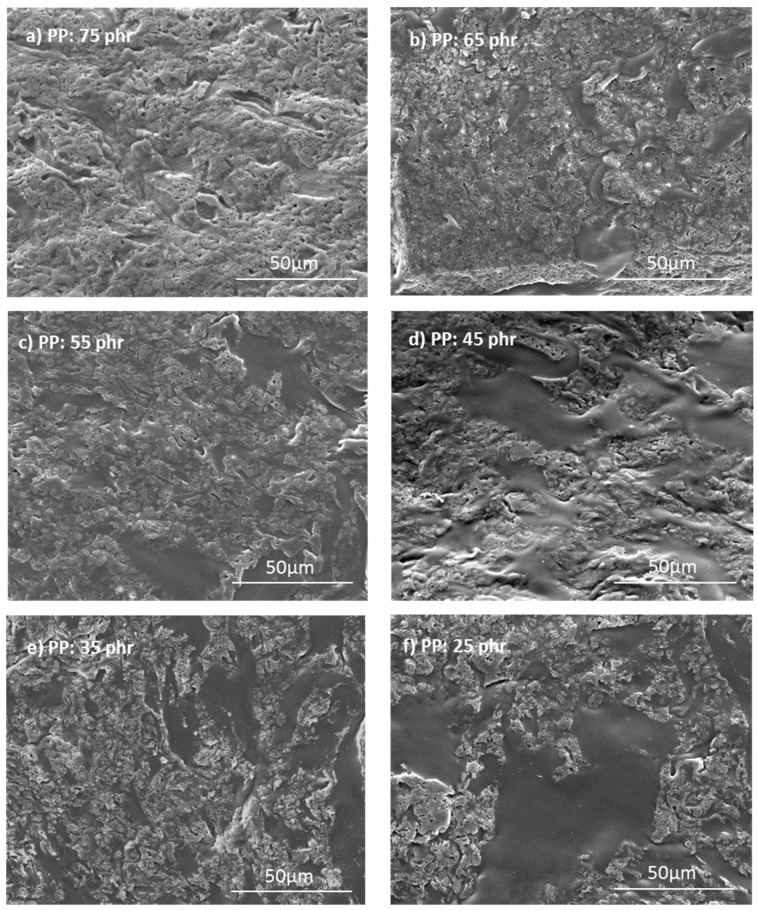
Evolution of the morphologies observed by SEM for TPV with different amounts of PP and crosslinked by DCP (4 phr).

The morphologies of the unfilled peroxide-based TPV were observed by SEM, and the images are shown in Figure 7. As expected, TPVs have a heterogeneous morphology in terms of the shape of both phases. In fact, it can be observed both large and small domains of crosslinked EPDM dispersed in the thermoplastic phase. As the PP concentration decreases, and thus the EPDM concentration increases, the larger EPDM domains predominate. In fact, as discussed in the introduction part, the concentration of EPDM is much higher (up to 80 wt %) than the maximum packing fraction we would have with spheres. These results show that only this type of morphology allows dispersing so much EPDM (60–80%) in PP from the phase inversion phenomenon.

**Figure 8:**
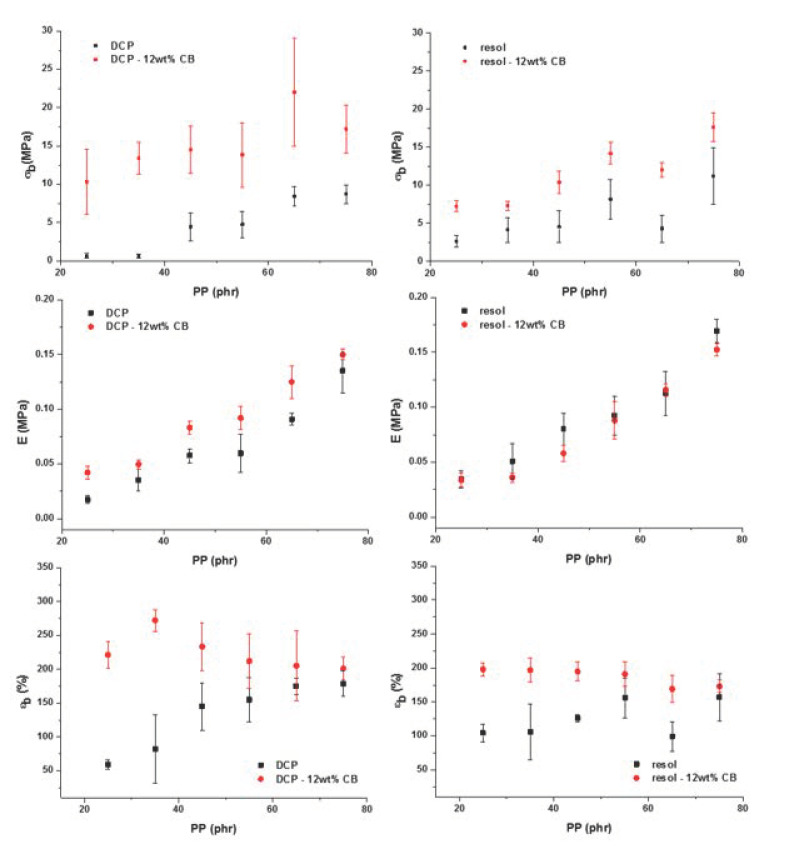
Evolution of the mechanical properties: tensile strength, Young modulus and elongation at break in function of the PP amount for unfilled and carbon black filled TPV.

However, when the PP concentration is decreased, a significant loss of the mechanical properties at break (stress and elongation) can be observed (See Figure 8). This result shows that the two phases (PP and EPDM) are not compatible as one might have expected although the interfacial tension between PP and EPDM is low (~0.3 mN/m) and the oil plasticizer also acts as a compatibilizer agent by favoring physical entanglements between PP and EPDM chains at the interface of both phases [6]. The decrease in Young modulus with the increase of the EPDM phase is expected because the TPV tends towards the Young modulus of pure crosslinked EPDM for the lowest amount of PP.

One strategy to compatibilize polymer blends that has been developed these last years is the addition of nanofillers [16]. Sumita et al. [19] proposed that the localization of CB particles can be determined by the wetting parameter: the nanofiller can be located in one phase or at the interface. Other studies [6,14] reported that the viscosity ratio of the two phases of the blend can also play a major role in the dispersion of the CB particles. Regarding PP/EPDM blends, few works have been devoted to their compatibilization from the addition on nanofillers. Yang et al. [20] performed a study on PP/EPDM/SiO_2_ nanocomposites by varying the amount of EPDM phase (10–30 wt %) and of the silica (1–5 wt %). Hydrophobic silica (particle size: 10–30 nm; S = 200 m^2^/g) and hydrophilic silica (particle size: 15–20 nm; S = 150 m^2^/g) were used. They showed that PP/EPDM/SiO_2_ systems exhibit the formation of a filler network structure in the PP matrix that lead to a super toughened ternary composite with Izod impact strength 2–3 times higher than PP/EPDM binary blends. The morphology of PP/EPDM blends at different proportions, especially at the co-continuity of both phases was studied by Martin et al. [21]. More particularly, they studied the influence of silica particles (hydrophilic or hydrophobic) on the co-continuous morphology. They observed that hydrophilic silica particles tend to migrate within the EPDM phase and form huge aggregates (0.5–1 μm). On the other hand, hydrophobic particles are dispersed homogeneously and can be found both at the interface and within the EPDM phase. The most interesting results have been obtained with carbon black [22,23,24] dispersed in TPV based on PP/EPDM. For example, Ma et al. [24] observed that with the addition of CB, the uncrosslinked EPDM/PP blend (Thermoplastic elastomer, TPE) and dynamically vulcanized blend (TPV) showed a notable difference in the conductive electrical properties, which is mainly caused by the different localization of CB particles resulting from the dynamic vulcanization process. Particularly, they found that the CB particles in the TPE were preferably dispersed in the EPDM phase, whereas the CB particles in the TPV were almost located in the PP matrix due to the high viscosity ratio of cured EPDM compared with PP. 

**Figure 9:**
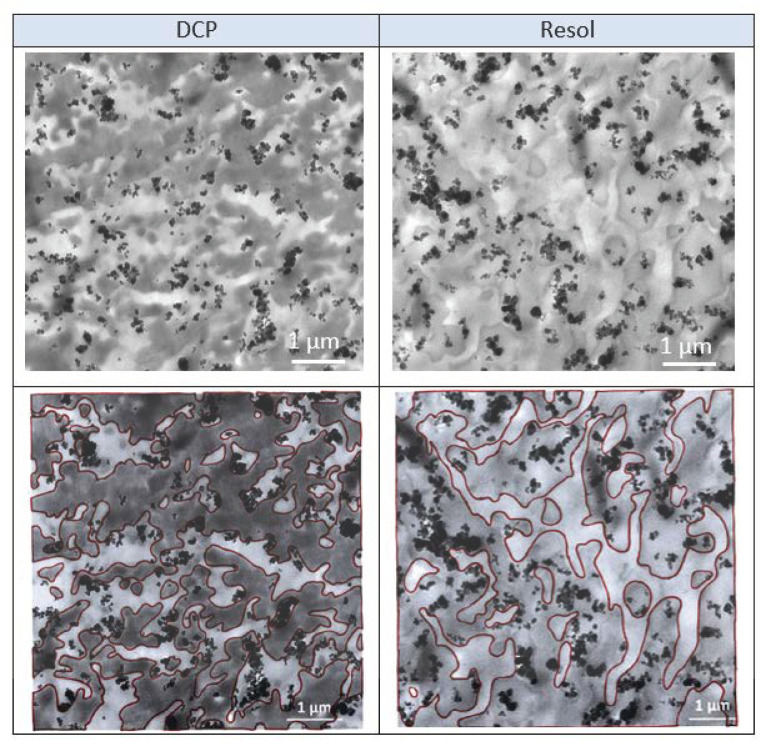
TEM pictures of filled TPV (PP: 75 phr) with 12 wt % of carbon black (**top**). Highlighting of phase limits (**bottom**) to help visualize the location of carbon black fillers.

In the present study, 12 wt % of carbon black was added in the TPV with the aim to improve the mechanical properties. According to the experimental protocol, CB was first mixed with EPDM/plasticizer before the addition of PP and the phase inversion phenomenon. From TEM pictures in Figure 9, it can be observed that the CB particles are located in the case of peroxide-based TPV at the interface between the PP and the EPDM, while the filler is located mainly in the EPDM phase in the case of Resol crosslinker. This result means that the carbon black localization is not governed by thermodynamic aspect such as wettability criteria or by viscosity aspects. Indeed, we have demonstrated in our previous work [18] that carbon black interacts with the chemical reaction of crosslinking due to the radical initiator and the chemical grafting of EPDM chains on the surface of CB can be expected.

Furthermore, a significant enhancement of tensile stress at 300% has been also reported with the addition of CB in TPV [23]. Actually, the mechanical properties can be then improved significantly when the fillers are located at the interface, because such a filler localization, like a compatibilizer, is helpful to refine the phase morphology and enhance interfacial adhesion.

The mechanical properties of filled TPV in Figure 8 show a significant improvement of the mechanical properties at break, more particularly for the lowest concentrations of PP. This improvement of the mechanical properties is even more important for peroxide-based TPV which can be explained with the localization of the CB at the interface between the two phases which enhance the interfacial adhesion. This phenomenon leads to a change in the morphology development and compatibilization. However, the phenomenon of carbon black localization is not well understood in such complex formulations.

## 4. Conclusions

The interesting feature of TPV is their ability to recover the elasticity measured by the compression set. The development of the morphology requires then the highest possible concentration in the crosslinked phase well above the maximum packing fraction of 0.64 for spheres. In order to achieve a high concentration of dispersed rubber phase, the EPDM phase must be dispersed with a large distribution of the domain size.

The main conclusions of the present work are the following:

The phase inversion between both phases takes place when the EPDM phase has reached a certain level of crosslinking (swelling ratio ~20 corresponding to a crosslinking density of 10 mol/m^−3^) which allows the stress applied by the PP matrix to disperse this phase by fragmentation. The maximal stress is observed at the melting temperature of the PP pellets. 

The crosslinking chemistry nature (radical initiator or phenolic resin crosslinker) have less influence on the elasticity recovery in the case of the TPV compared with a pure EPDM system. This result means that the morphology plays an important role in the compression set of TPVs. Morphology is the first order parameter whereas crosslinking density of the EPDM phase is a second order parameter.

The morphologies of the peroxide-based TPV, observed by SEM, show heterogeneous systems with large EPDM domains. However, this large distribution of EPDM particles is the only way to increase the maximal packing fraction of crosslinked EPDM phase which behave as solid particles.

The addition of carbon black fillers allows the mechanical properties at break to be significantly improved at the lowest PP concentration (20%). This improvement of mechanical properties is still not well understood.

Finally, to reach the best compression set while keeping good mechanical properties, a peroxide-based TPV containing carbon black and the less PP as possible should be formulated. Therefore, the low quantity of PP allows to have a good compression set; the peroxide permits the carbon black to be located at the interface between the two phases and thus the filler can reinforce the mechanical properties of the material.

## Figures and Tables

**Table 1:** Formulations of samples prepared in the batch mixer according to the mixing process described in Figure 3 (in phr).

	EPDM	Oil	PP	DCP	Resol	CB
**Variation of the crosslinking agent**	100	122	75	1	-	-
	100	122	75	2.75	-	-
	100	122	75	4	-	-
	100	122	75	5.5	-	-
	100	122	75	6.5	-	-
	100	122	75	-	1	-
	100	122	75	-	2.75	-
	100	122	75	-	4	-
	100	122	75	-	5.5	-
	100	122	75	-	6.5	-
**Variation of the PP content**	100	122	75	4	-	-
	100	122	65	4	-	-
	100	122	55	4	-	-
	100	122	45	4	-	-
	100	122	35	4	-	-
	100	122	25	4	-	-
	100	122	75	-	7	-
	100	122	65	-	7	-
	100	122	55	-	7	-
	100	122	45	-	7	-
	100	122	35	-	7	-
	100	122	25	-	7	-
**Addition of CB filler**	100	122	75	4	-	41
	100	122	65	4	-	39
	100	122	55	4	-	38
	100	122	45	4	-	37
	100	122	35	4	-	35
	100	122	25	4	-	34
	100	122	75	-	7	41
	100	122	65	-	7	39
	100	122	55	-	7	38
	100	122	45	-	7	37
	100	122	35	-	7	35
	100	122	25	-	7	34

**Table 2:** Values of compression set for TPV based on a decreasing concentration of the PP phase. Amount of crosslinker in the formulations: 4 phr (DCP) and 7 phr (Resol). Amount of CB: 12 wt %.

DCP (4 phr)			Resol (7 phr)		
PP (phr)	Cs (%) Unfilled Samples	Cs (%) Filled Samples	PP (phr)	Cs (%) Unfilled Samples	Cs (%) Filled Samples
75	56 ± 2	50 ± 2	75	56 ± 1	55 ± 1
65	45 ± 4	45 ± 5	65	49 ± 2	51 ± 2
55	41 ± 2	40 ± 2	55	42 ± 1	43 ± 3
45	34 ± 1	37 ± 3	45	39 ± 0	40 ± 1
35	32 ± 5	33 ± 2	35	36 ± 3	37 ± 2
25	30 ± 1	29 ± 2	25	32 ± 2	33 ± 1
